# A pH-sensitive liposomal co-delivery of fingolimod and ammonia borane for treatment of intracerebral hemorrhage

**DOI:** 10.1515/nanoph-2022-0496

**Published:** 2022-11-02

**Authors:** Xiyu Gong, Xingyu Fan, Yongju He, Yingwei Wang, Fangfang Zhou, Binbin Yang

**Affiliations:** Department of neurology, The Second Xiangya Hospital, Central South University, Changsha, Hunan 410011, China; School of Materials Science and Engineering, Central South University, Changsha, Hunan 410083, China; Hunan Key Laboratory of Nanophotonics and Divices, Central South University, Changsha, Hunan 410083, China

**Keywords:** ammonia borane, fingolimod, intracerebral hemorrhage, liposome

## Abstract

Intracerebral hemorrhage (ICH) is one of the most devastating types of stroke. This study aims to develop a new drug carrier with hematoma-specific response and high property. pH-sensitive liposomes (PSL) were developed. Fingolimod with ammonia borane were encapsulated in the phospholipid vesicles to integrate two drugs for treating ICH more effectively. pH sensitive PSL-FTY720/AB was characterized for various physicochemical parameters such as shape, surface morphology, vesicle size, zeta-potential, *in-vitro* drug release in different pH environment, cellular toxicity, *in-vivo* and ex-vivo tissue accumulation. *In vivo* results further confirmed that drug-loading nanoparticles effectively protected against ICH-induced brain injury through synergistic effect of anti-inflammation and anti-oxidation. Collectively, the present study confirmed that PSL-FTY720/AB can be an effective, safe, and a novel alternative treatment approach in ICH.

## 1 Introduction

ICH accounts for about 15% of all strokes and culminates in high mortality and disability [1]. Brain injury following ICH is initiated by the mechanical damage caused by the initial hematoma [2], which is closely followed by secondary brain injury (SBI) [3]. There is emerging evidence that oxidative stress and inflammation are closely associated with SBI after ICH [4, 5], and that the interactions between them may play critical roles in SBI. Oxidative stress induces inflammation, while inflammation causes damage through oxidative stress [6]. The malignant positive feedback loop contributes secondary clinical deterioration. Hence, co-delivery of anti-inflammation and anti-oxidative agents can exert synergistic effects after ICH.

Fingolimod (FTY720) is approved oral therapy for multiple sclerosis (MS) [7]. It not only reduces inflammatory damage but also promotes neuroprotection [8]. Recently, clinical studies showed that Fingolimod effectively reduces SBI after ICH by modulating systemic inflammation and protecting vascular permeability [9, 10]. On the other hand, as a rising approach, H2 therapy is now recognized as a therapeutic antioxidant in various diseases [11, 12]. However, the current H2 treatment modalities rely predominantly on the systemic administration of the gas, which is related to poor targeting and utilization [11]. Recently, scholars have realized that ammonia borane(AB) is a safe and feasible solid hydrogen storage material due to its nontoxicity, high hydrogen content, and high solubility in water [13, 14].

As BBB serves as a significant impediment to the delivery of therapeutic agents to disease-affected brain tissue, many agents are forced to be administered in an invasive manner [15]. BBB integrity can become compromised after various insults, however, the extent of permeability changes are not sufficient to permit adequate drug delivery into brain [16]. Moreover, many peptides and small-molecule drugs can be quickly degraded and eliminated during circulation, and hence pharmacologically sufficient concentration and long-lasting effects cannot be guaranteed. In recent years, nano delivery systems provide target-oriented delivery and controllable release of drugs [17, 18]. Liposomes are stable nanosystems with unique phospholipid bilayer structure, which facilitate the permeation of drug across various biological membranes [19]. Liposomes are widely utilized as drug carriers due to good biocompatibility, nontoxic and biodegradable, high delivery efficiency, and versatile structure modification [20]. Moreover, liposomes can be produced with appropriated size ranging from 50 nm to 150 nm, with an enhanced permeability and retention(EPR) effect [21]. However, liposome itself does not specifically accumulate in ICH sites. Specific nano-materials are used to modify the liposomes. The development of pH-sensitive liposomes for ICH treatment in our study is based on the fact that mitochondrial dysfunction [22, 23] and intracellular Ca2+ overload [24] after ICH may induce cerebral energy failure and local acid environment. pH-sensitive liposomes were designed to be stable at physiological pH, but to be destabilized upon acidification by SBI after ICH, which promote the release of their encapsulated contents.

In the current study, we constructed a novel pH-sensitive liposome encapsulating FTY720 and AB(PSL-FTY720/AB). The *in vivo* targeting efficiency and neuroprotective effect were determined in a murine model of ICH.

## 2 Materials and methods

### 2.1 Materials

Soy phosphatidylcholine (SPC) was ordered from Taiwei Pharmaceutical Co., Ltd. (Shanghai, China). Cholesterol (Chol) was purchased from Huixing Biochemical Reagent Co. Ltd (Shanghai, China). DOPE was purchased from Sigma Aldrich Co. Ltd (St. Louis, MO, USA). DSPE-PEG-COOH was purchased from Sigma Aldrich Co. Ltd (St. Louis, MO, USA). 3-(4,5-dimethylthiazol-2-yl)-2,5-diphenyltetrazo-liumbromide (MTT) were offered by Sunshine Biotechnology Co., Ltd (Nanjing, China). FTY720 was purchased from Sigma Aldrich Co. Ltd (St. Louis, USA). NH_3_BH_3_ was purchase from Sigma Aldrich Co. Ltd (St. Louis, USA). Fetal bovine serum (FBS, Hyclone^®^) and Dulbecco’s modified Eagle medium (DMEM, Hyclone^®^) were purchased from SunShine Biotechnology Co. Ltd (Nanjing, China). Evens blue was provided by Sigma Aldrich Co. Ltd (St. Louis, USA). DAPI was provided by Beyotime Institute of Biotechnology Co. Ltd (Nantong, China). Human neuroblastoma cells (SH-SY5Y) were purchased from American Type Culture Collection. C57BL/6 mice were purchased from Slack Jingda Co. Ltd (Changsha, China).

### 2.2 Synthesis and characterization of pH-sensitive FTY720-AB lip

First, DOPE: DSPE-PEG-COOH: cholesterol: SPC prepared at the ratio of 6:6:5.2:4, with or without ammonia borane (NH3BH3, AB, 1 mg) were dissolved in chloroform. To eliminate chloroform from the lipids and lipidic film preparation, the film was flushed with nitrogen gas and further dried by storing in the flask overnight in a vacuum desiccator. Next, FTY720 solution (10 mL, 10 mg/mL) was added. The mixture of aquatic and lipidic phases were vigorously vortexed for 5 min and then sonicated for 45 s at 45 °C under argon gas exposure. Finally, PSL-FTY720/AB was obtained by Liposome high pressure homogenizer.

The morphology of PSL-FTY720/AB was imaged with transmission electron microscope (TEM, JEM-2100F). The hydrodynamic diameter size and zeta potential of nanoparticles were examined by a dynamic light scatter analyzer (Zetasizer Nano Z90, Malvern). Encapsulation efficiency (EE) of FTY-720 was determined by HPLC (Agilent, USA). Encapsulation efficiency (EE) of AB was determined by color removal of methylene blue. %Drug Entrapment = Amount of entrapped drug/Total drug added × 100%

### 2.3 In vitro drug release

The drug release of FTY720 was investigated in acetate solutions at different pH values (pH 7.4, 6.5, 5.5, 4.5) by dialysis. Briefly, liposomes (2.285 mg/mL, 600 μL) were transferred into dialysis bag and then added in 5 mL of acetate solution (37 °C, stirred at 100r/min). At pre-arranged time intervals, 2 mL of release media was extracted to evaluate the concentration of FTY720 through spectrophotometer, and equal volume of acetate solution was resupplied. The drug release of hydrogen was detected using the reduction of methylene blue (pH 4.5).

### 2.4 Cell culture and animal care

Human neuroblastoma (SH-SY5Y) cells were cultured in Dulbecco’s modified Eagle medium and Ham’s F-12 (DMEM/F12) with high glucose and 10% FBS under an atmosphere of 5% CO2 at 37 °C in an incubator (Thermo Scientific, USA). This study was approved by the Animal Ethics Committee of the Second Xiangya Hospital of Central South University in compliance with NIH guidelines. Male C57BL/6 mice with weight from 25 g to 28 g and about 8–10 weeks age old were employed in our study.

### 2.5 Cytotoxicity in vitro

Vitro cytotoxicity was investigated using methylthiazoletetrazolium (MTT) [3-(4, 5-dimethylthiazol-2-yl)-2, 5-dephenyl-2H-tetrazolium bromide] assay. Briefly, SH-SY5Y cells were seeded in 96-well plates and cultured for 24 h. The cells were then incubated with gradient concentration of FTY720, PSL-FTY720, PSL-FTY720/AB, respectively, for another 24 h. Subsequently, MTT solution (5 mg/mL) was added to each well and the plates were incubated at 37 °C. After 4 h incubation, the medium was removed. Dimethyl sulfoxide (DMSO) was used to dissolve the internalized crystals. Absorbance was detected using a microplate reader (Thermo scientific, USA). The cellular viability was evaluated through normalized to control.

### 2.6 Establishment of an ICH mouse models and animal groups

Mice were anaesthetized with 2% pentobarbital sodium (40 mg/kg). A burr hole was undertaken by a dental drill (2 mm lateral to bregma). 0.0375U in 0.5 μL collagen type IV was slowly injected into the left hemisphere at 3.75 mm deep below the skull surface by micro pump for 5 min on a stereotaxic frame. The needle was then maintained for 10 min to prevent reflux. C57BL/6 mice were divided into four groups: ICH group (normal saline + ICH), free FTY720 + ICH (FTY720 group), the PSL-FTY720 group + ICH (PSL-FTY720/AB group) and the PSL-FTY720/AB + ICH(PSL-FTY720/AB group). The mice were intravenously injected via tail vein with FTY720, PSL-FTY720 and PSL-FTY720/AB (0.5 mg/kg, dose equivalent to FTY720) once a day until sacrifice.

### 2.7 Biodistribution

In the present study, mice suffered from ICH were employed to evaluate *in vivo* biodistribution of PSL-FTY720/AB. A near-infrared fluorophore dye (DiR)-loaded nanoparticles were prepared as described previously to obtain a final DiR concentration of 50 μg/mL. C57BL/6 mice subjected to ICH were injected intravenously with PSL^Dir^-FTY720/AB (0.4 mg/kg). After 2, 8, and 24 h, the mice were anesthetized with 10% chloral hydrate (i.p.) and observed using vivo imaging system of IVIS Lumina III (Perkin Elmer, USA) with fluorescent filter sets (excitation:748, emission:780 nm). Thereafter, the mice were sacrificed and the major organs (heart, brain, liver, spleen, and kidney) were excised for *in vivo* imaging.

### 2.8 Behaviour test

The neurological deficit scores were determined by extensively used modified neurological severity score (mNSS, *n* = 8) 1, 3 and 7 days after ICH. The mNSS is ranged from 0 to 18 point. The higher point is associated with the more severe disability. The mNSS included motor, sense, reflect and muscular tension test.

### 2.9 Brain water content

Brain water content was investigated as previous study. Briefly, mice (*n* = 6) were sacrificed after anesthesia. Brain was cut into 4 mm coronal slice. The brain slice was divided into five parts: contralateral and ipsilateral basal ganglia, contralateral and ipsilateral cortex, and cerebellum. Each part was obtained wet weight (WW) and then dried at 100 °C for 72 h to evaluate the dry weight (DW). Brain water content was calculated as follows: [(WW − DW)/WW] × 100%.

### 2.10 Evans blue leakage

To determine the permeability of the BBB, evens blue (2%, 4 mg/kg, Sigma, USA) was injected into femoral vein at 72 h after ICH. After 2 h, mice (*n* = 4) were deeply anesthetized and perfused with saline. Brains were quickly obtained, weighted and then homogenized. The supernatant after centrifugation was collected and diluted with ethanol. The absorbance was detected with a spectrophotometer and evaluated through a standard curve (Thermo Fisher Scientific, USA).

### 2.11 HE staining

Main organs (brain, heart, kidney, liver, lung and spleen) (*n* = 3) were fixed in 4% PFA overnight. Tissues were then embedded into paraffin and cut into 5 µm thick sections. Tissue sections were dewaxed and rehydrated through gradient ethanol into water. Thereafter, the sections were immersed in a Hematoxylin solution. After a water-rinse, sections were stained with eosin for 3 min and dehydrated in a graded series of alcohol and xylene. They were investigated under microscope (Olympus, Japan).

### 2.12 Nissl and FJB staining

For Nissl staining, brain sections were stained with toluidine blue after dewaxation and then dehydrated with alcohol and blocked with neutral gum. For FJB staining, brain sections were incubated with potassium permanganate for 20 min and washed with distilled water. Sequentially, the sections were submerged in FJB solution. The sections were observed under fluorescence microscope (Olympus, Japan).

### 2.13 Dihydroethidium staining

Dihydroethidium (DHE) fluorescence staining was applied to measure intracellular ROS production after ICH. Briefly, frozen sections (10 μm, *n* = 3) of brain tissues were incubated with dye DHE. DHE-positive cells were observed with a fluorescence microscope (Olympus, Japan).

### 2.14 ELISA

Expression of inflammation markers, including TNF-α and IL-1β, from perihematomal tissue extracts was measured by respective ELISA kits (R&D Systems), according to manufacturer’s instructions.

### 2.15 Statistical analysis

Statistical analysis was used SPSS 21software. All data were shown as mean ± SD Distribution of the data was evaluated with the Kolmogorov-Smirnov test. Statistical differences were analyzed with one-way ANOVA as the data followed normal distribution, otherwise with Kruskal–Wall H. The threshold for significance was *P* < 0.05.

## 3 Results

### 3.1 Synthesis and characterization of drug-loaded nanoparticles

**Figure 1:**
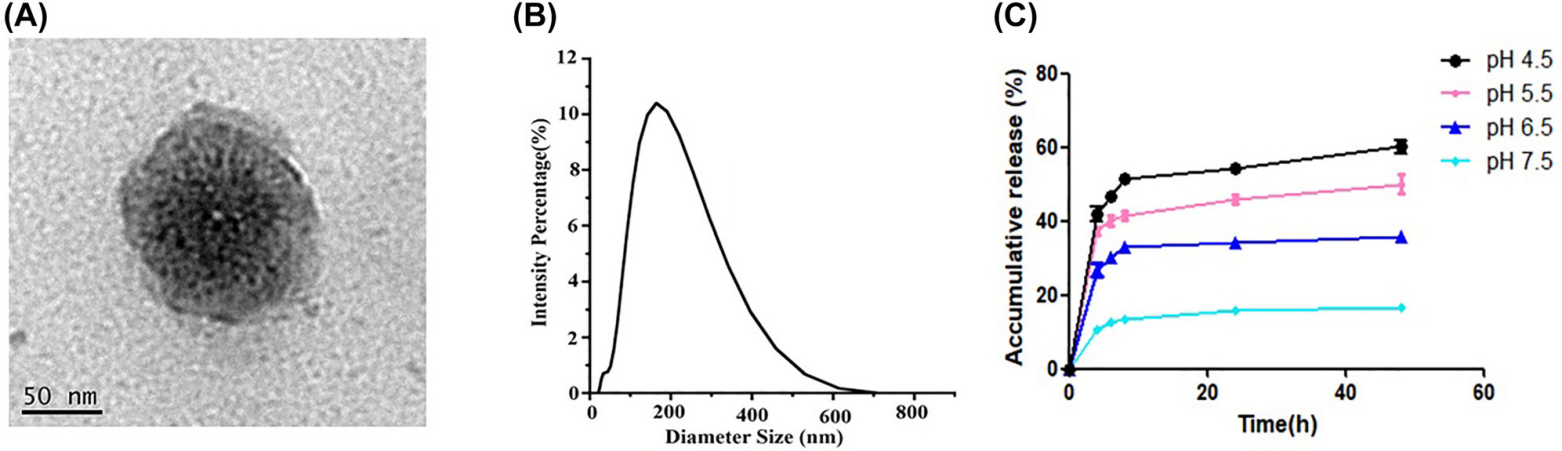
TEM images of PSL-FTY720/AB (A), dimeter of PSL-FTY720/AB (B), and vitro release of FTY720 at different pH value (C).

To obtain PSL-FTY720/AB, we applied DOPE as lipid bilayer. PEGylated PSL-FTY720/AB showed increased their blood-circulation time and presented as well-dispersed uniform nanospheres when imaged by TEM (Figure 1A). Most drug-loaded nanoparticles were generally spherical, separated, and homogeneously distributed, indicating stable formulations. The nanoparticle diameter size was about 145 nm (Figure 1B) and the zeta potential was −28.33 ± 1.68 mV at pH 7.4 measured by dynamic light scattering. The encapsulation efficiency of FTY720 and hydrogen gas was around 33% and 0.27 μmol/mg, respectively.

### 3.2 In vitro drug release of drug-loaded nanoparticles

PSL-FTY720/AB showed a slightly higher FTY720 release at endo-lysosomal pH (5.5–6.5) during the first 4 h incubation than at pH 7.5, and a notably stable release behavior after 8 h, which reached a cumulative release amount of 35–55% at 72 h. In comparison, at 72 h of incubation, only 14.75% ± 9.34% FTY720 were cumulatively released at pH 7.5 (Figure 1C).

### 3.3 Cytotoxicity of drug-loaded nanoparticles

**Figure 2:**
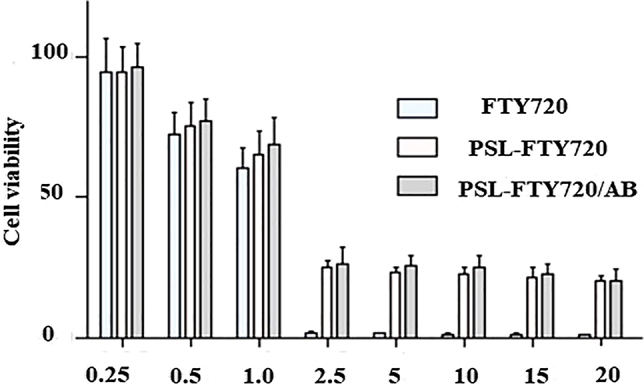
*In vitro* cytotoxicity of FTY720, PSL-FTY720 and PSL-FTY720/AB at pH 7.5 for 24 h.

MTT assay was performed to evaluate the cytotoxicity of PSL-FTY720/AB to SHSY-5Y cells. SHSY-5Y cells were incubated with gradient concentration of free FTY720, PSL-FTY720 and PSL-FTY720/AB at the same equivalent FTY720 concentration at pH 7.4, respectively. Cell viability was measured after 24 h. With low-dose treatment, SHSY-5Y cells showed high viability in cases of free drug and nanoformulations. However, all drugs produced cellular toxicity when used at high doses (>2.5 μg/mL). Notably, MTT assay demonstrated significantly higher cytotoxicity of FTY720 over PSL-FTY720 and PSL-FTY720/AB, respectively. The results indicated that the pH-sensitive liposome effectively reduced the cytotoxicity (Figure 2).

### 3.4 Biosafety assessment of drug-loaded nanoparticles *in vivo*

**Figure 3:**
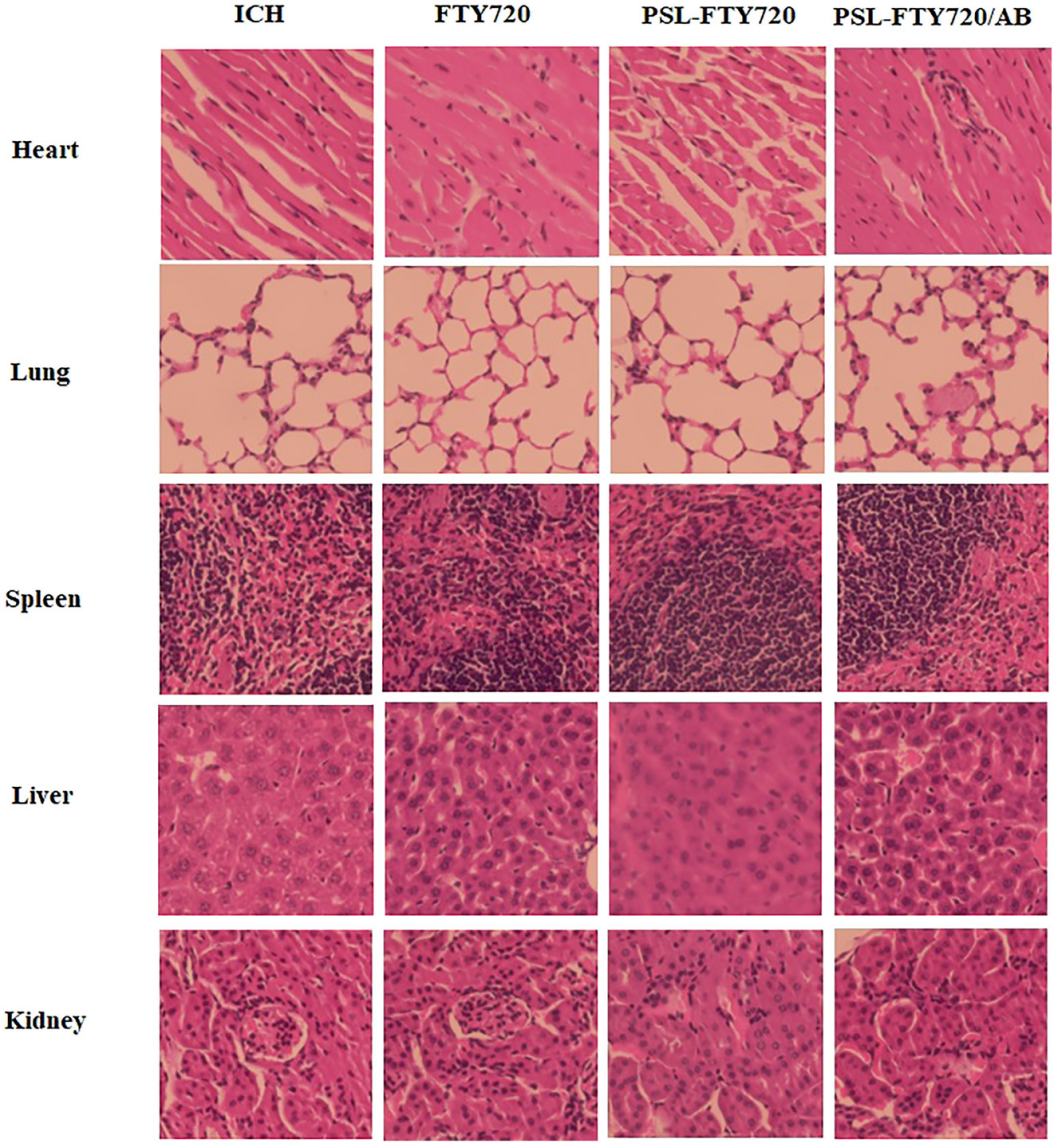
H&E staining of main organs after administration of FTY720, PSL-FTY720 and PSL-FTY720/AB.

Toxicity studies were also performed in mice. The drugs were injected intravenously into mice. After 3 days, various organs (heart, liver, spleen, lung and kidney) of mice were removed and observed with H&E staining. Compared with the control group, there were no significant pathological changes in normal tissues in PSL-FTY720/AB group (Figure 3). These results showed that application of PSL-FTY720/AB was feasible and safe.

### 3.5 *In-vivo* and ex-vivo biodistribution

**Figure 4:**
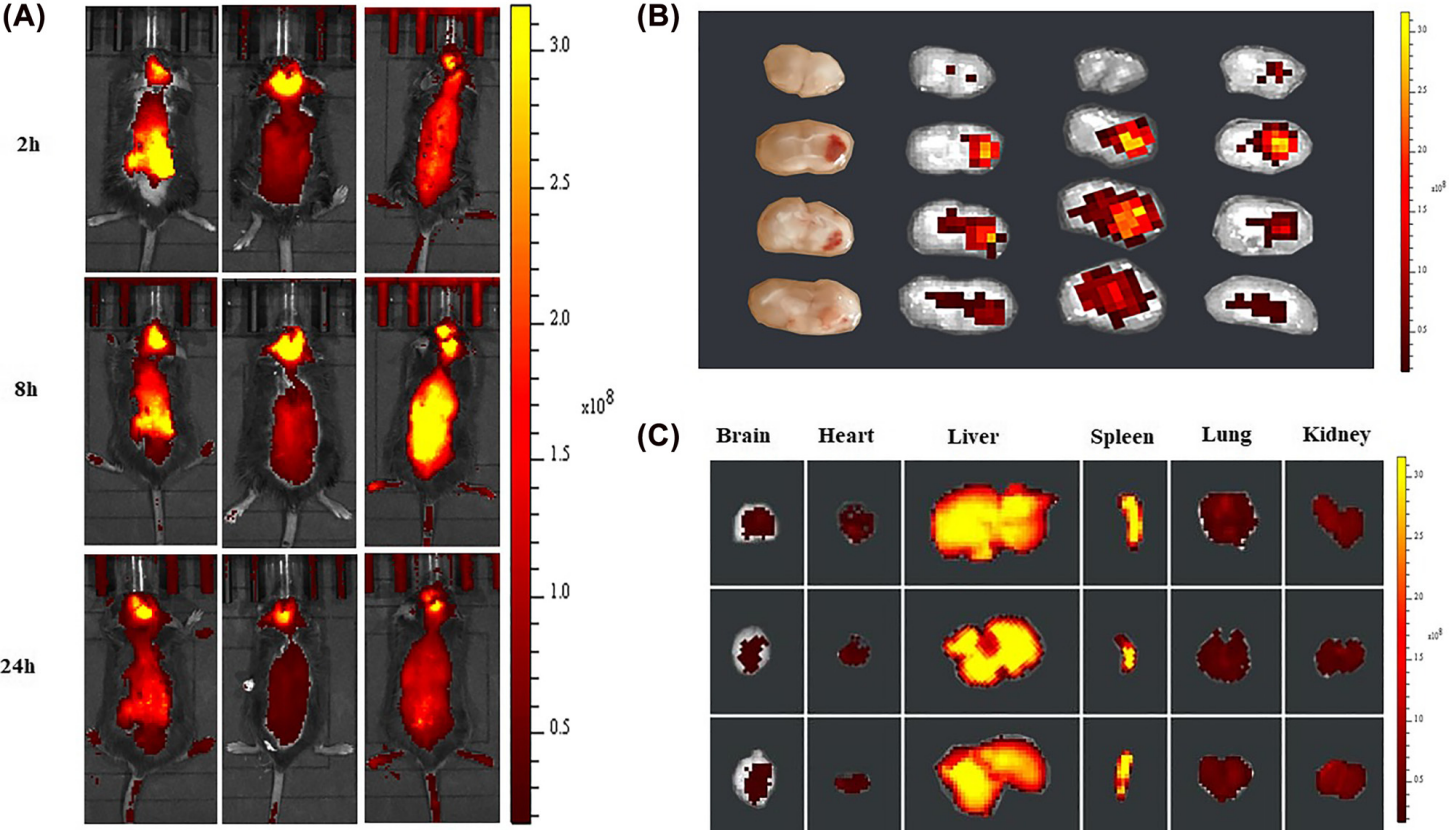
*In vivo* imaging of biodistribution of DIR contained PSL-FTY720/AB (*n* = 3) at different time points after intravenous injection into the mice subjected to ICH (A). Accumulation of DIR-labeled PSL-FTY720/AB in the brain and different organs were detected using the ex vivo imaging at 24 h post-injection (B and C).

The real-time fluorescence images of PSL^DIR^-FTY720/AB *in vivo* demonstrated that PSL^DIR^-FTY720/AB rapidly trafficked into brain tissue within 2 h. The targeting behavior of PSL^DIR^-FTY720/AB reached a peak at 8 h and maintained until 24 h (Figure 4A). Mice were sacrificed after 24 h and the main organs (brain, heart, lung, liver, spleen and kidney) were dissected for ex vivo imaging. The cryosection of brain tissues of mice post-ICH were conducted to examine the brain distribution of PSL-FTY720/AB. Dir-labeled-Lip mainly accumulated in hematoma and perihematomal area ex vivo (Figure 4B). The distribution of liposomes in major organs was also determined. PSL^DIR^-FTY720/AB mainly accumulated in the liver and spleen (Figure 4C). These results revealed that PSL^DIR^-FTY720/AB can penetrate the BBB rapidly through blood circulation and deliver drugs to the targeted brain tissues.

### 3.6 PSL-FTY720/AB ameliorated ICH-induced neurological deficits

**Figure 5:**
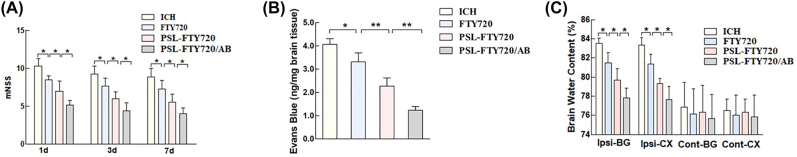
Assessment of neurological defects in mice at 1d, 3d and 7d after ICH (A), and examination of Evans blue (B), and brain water content (C) in mice 3d after ICH.

The mice were intravenously injected with saline, FTY720, PSL-FTY720/AB or PSL-FTY720/AB (FTY720-equivalent dose 0.5 mg/kg) immediately after ICH once a day until sacrifice. The mNSS neurological function score was performed at 1d, 3d, and 7d after ICH to evaluate the neurological function recovery of the mice. The mNSS scores of FTY720, PSL-FTY720 and PSL-FTY720/AB treatment groups were considerably lower than that of the ICH group. Among all treatment groups, PSL-FTY720/AB exhibited the best efficacy in alleviating neurological deficits (Figure 5A). These results suggested greater therapeutic efficacy with PSL-FTY720/AB than with FTY720 and PSL-FTY720.

### 3.7 PSL-FTY720/AB reduced brain edema and rescued BBB disruption

Brain water content was evaluated in contralateral and ipsilateral basal ganglia and cortex, and cerebellum of each group. Consistent with the behavioral results, FTY720, PSL-FTY720 or PSL-FTY720/AB significantly reduced the water content in the ipsilateral basal ganglia and cortex at 72 h post-ICH; PSL-FTY720/AB group had a relatively slighter brain edema than other two group (Figure 5B). There were no differences in brain water content observed between all groups in contralateral cortex, contralateral basal ganglia or in cerebellum. To evaluate the BBB integrity, EB staining was performed at 72 h after ICH. Consistent with the findings of brain edema, the EB concentration in the brain significantly differed among the four groups. EB leakage was considerably lower in FTY720, PSL-FTY720 or PSL-FTY720/AB groups than that in the ICH group. PSL-FTY720/AB group still had a significant lower EB leakage than other two groups (Figure 5C).

### 3.8 PSL-FTY720/AB reduced ICH-induced brain damage

**Figure 6:**
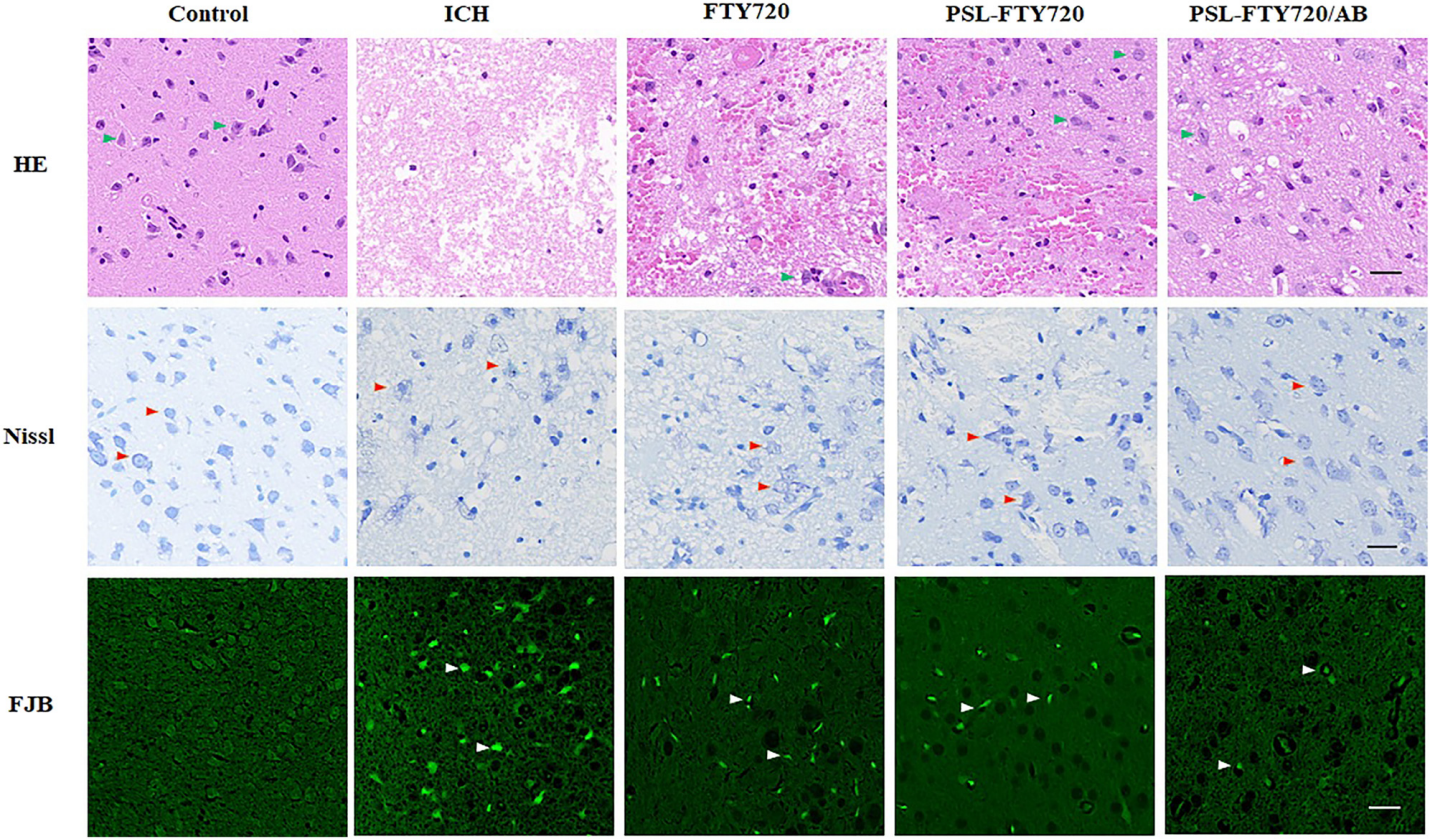
Assessment of HE, Nissl and FJB staining in perihematomal tissue at 3d after ICH.

We applied HE staining to assess the histopathological changes in the cortex. Neurons from the sham group showed normal morphology, which were neatly arranged with dense cytoplasm and clearly visible nuclei. The ICH group showed a neuronal loss compared with sham group at 72 h after ICH. Moreover, irregular arrangement, disorganization, and karyopyknosis of the neuronal cells were identified. The mice from FTY720, PSL-FTY720 and PSL-FTY720/AB group exhibited more neuronal quantity and clearer cell contours after ICH. Among three treatment groups, PSL-FTY720/AB showed most significant inhibition of neuronal apoptosis. The results of Nissl staining were similar. In comparison with the ICH groups, the number of atrophic neurons exhibiting damaged nuclei and shrunken cytoplasm decreased in FTY720, PSL-FTY720 and PSL-FTY720/AB group. FJB staining also showed that the number of degenerated neurons in FTY720, PSL-FTY720 or PSL-FTY720/AB group was less than that in ICH group, with PSL-FTY720/AB produced the most significant results (Figure 6). Generally, FTY720, PSL-FTY720 and PSL-FTY720/AB groups reduced the severity of the cerebral injury. Compared to FTY720 and PSL-FTY720 treatment, PSL-FTY720/AB treatment had the most significant effect on neuronal apoptosis.

### 3.9 PSL-FTY720/AB treatment decreased ROS production

**Figure 7:**
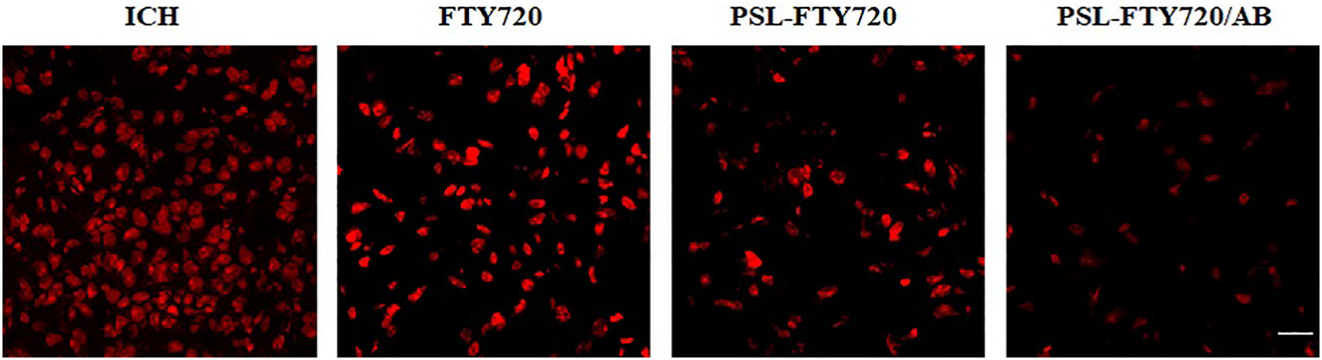
Assessment of DHE staining in perihematomal tissue at 3d after ICH.

Excessive ROS production during ICH leads to oxidative stress, an important pathogenic factor of SBI. DHE was used as a fluorescent probe for the detection of ROS generation. FTY720, PSL-FTY720, and PSL-FTY720/AB substantially decreased the number of DHE positive cells compared to ICH group. There were significant differences among FTY720, PSL-FTY720, and PSL-FTY720/AB groups. PSL-FTY720/AB had more profound inhibition effect on ROS production than the other two treatments (Figure 7).

### 3.10 PSL-FTY720/AB decreased pro-inflammatory cytokines production

**Figure 8:**
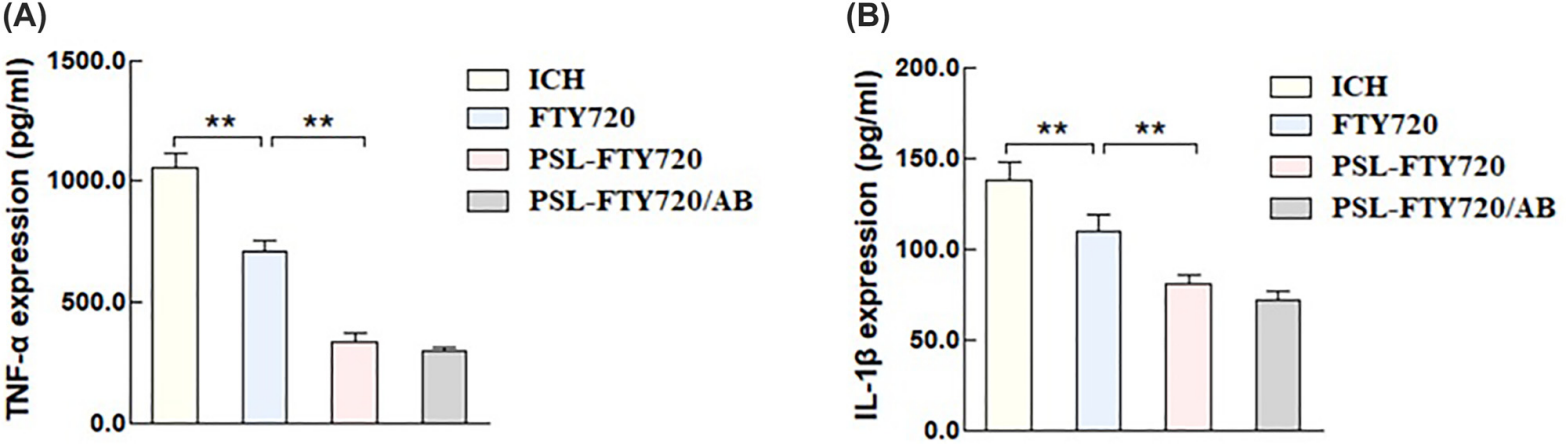
TNF-α (A), and IL-1β (B) in perihematomal tissues were quantified by ELISA.

To evaluate the effect of PSL-FTY720/AB on neuroinflammatory response, pro-inflammatory cytokines, including tumor necrosis factor-α (TNF-α) and interleukin-1β (IL-1β), in perihematomal tissue were quantified by ELISA. The results showed that FTY720, PSL-FTY720, and PSL-FTY720/AB reduced inflammatory cytokine concentrations compared with ICH group. PSL-FTY720/AB had similar effect with PSL-FTY720, which led to significant reduction of TNF-α and IL-1β compared with FTY720 (Figure 8A and B).

## 4 Discussion

In this study, we encapsulated FTY720 and ammonia borane in pH-sensitive liposomes(PSL-FTY720/AB) for the enhanced penetration across BBB, ICH area targeting, and limited side effects during the blood circulation. The drug-carrying pH-sensitive liposomes were self-assembled nanoparticles, which had uniform spherical shape with uniform particle size, good dispersion, and no apparent aggregation. The particle size detected by Zetasizer Nano showed an average hydrodynamic diameter 145 nm. It was interesting to note that PSL-FTY720/AB in PBS had a negative zeta potential of −28.33 ± 1.68 mV at pH 7.4. The negatively charged vesicles are reported to be less cytotoxic or completely safe when compared to their cationic counterparts [25, 26]. Furthermore, it has been postulated that anionic vesicles, in general, associate more efficiently and are taken up more readily by the cells compared with neutral or zwitterionic vesicles [27]. Evaluation of toxicity of PSL-FTY720/AB in both *in vivo* and *in vitro* experiments indeed verified that the nanoparticles effectively reduced the cytotoxicity of the drug.

After ICH, hematoma mass effect leads to energy metabolism depletion in the surrounding hypoxic-ischemic tissue. ATP deficiency and consequent reduction of various ATPase activities [28] consequently result in a decreased extracellular pH in perihematomal area. According to the results we obtained, the drop in pH increased the release of drugs encapsulated in nanoparticle. The *in vivo* and ex vivo results confirmed that PSL-FTY720/AB could be effectively penetrated BBB and enriched in hematoma and perihematomal site in the mice. Collectively, we used pH-sensitive liposomes as platforms to design nanocarriers with low cytotoxicity, which were suited for drug delivery across the BBB and targeting perihematomal area.

The pathological mechanisms of hematoma after ICH, including oxidative stress and inflammatory responses trigger a series of adverse events causing SBI, and subsequent severe neurological deficits [29]. Oxidative stress and inflammation have been proved to be interrelated and interacted, forming a complicated pathway of SBI after ICH, including blood-brain barrier (BBB) destruction, brain edema, and brain injury [30–32]. Therefore, how to scavenge ROS and down-regulate inflammatory cytokines has been a critical strategy for the therapy of ICH. Current laboratory and clinical data suggest a beneficial role of FTY720 in ICH, which reduces neuroinflammation [33]. H2 therapy as a therapeutic antioxidant can attenuate the oxidative stress of major diseases [34]. In addition, over the recent past, ammonia borane (NH3BH3, AB) has attracted increasing attention as one of the most fascinating hydrogen storage materials because of its potential to store a significant percent of hydrogen chemically and low molecular weight [35]. In this work, we found that PSL-FTY720/AB treatment attenuated the neurological behavior impairment of mice subjected to ICH, as well as mitigated BBB destruction and neuronal loss induced by ICH. The significant lower ROS levels and decreased level of proinflammatory cytokines (TNF-α and IL-1β) in PSL-FTY720/AB group indicated that PSL-FTY720/AB exerted a synergistic effect to inhibit neuroinflammation and oxidative stress after ICH. In a word, PSL-FTY720/AB would be a promising therapy for ICH with attenuated toxicity and improved efficacy.

## 5 Conclusions

In this work, we designed a pH-sensitive liposome encapsulating fingolimod with ammonia borane (PSL-FTY720/AB). PSL-FTY720/AB effectively inhibited ICH-induced brain-injury-related inflammation as well as oxidative stress, and thus played a neuroprotective role after ICH.
